# Crosstalk in the diseased plasma cell niche – the force of inflammation

**DOI:** 10.3389/fimmu.2023.1120398

**Published:** 2023-02-21

**Authors:** Anna-Lena Schäfer, Paola Fernanda Ruiz-Aparicio, Antoine N. Kraemer, Nina Chevalier

**Affiliations:** ^1^ Department of Rheumatology and Clinical Immunology, Medical Center - University of Freiburg, Faculty of Medicine, University of Freiburg, Freiburg, Germany; ^2^ Center for Chronic Immunodeficiency (CCI), Medical Center - University of Freiburg, Faculty of Medicine, University of Freiburg, Freiburg, Germany; ^3^ Spemann Graduate School of Biology and Medicine (SGBM), University of Freiburg, Freiburg, Germany

**Keywords:** plasma cell, inflammation, plasma cell niche, autoimmunity, microenvironment

## Introduction

Persistence for years after antigen exposure is one hallmark of memory plasma cells (PCs) mediating long-term protection against pathogens. A crucial factor for this is the retention in survival niches, providing extrinsic factors regulating PC maturation and survival. Under homeostatic conditions, the bone marrow (BM) and intestinal lamina propria represent typical locations for memory PCs (1–7). However, PC niches can additionally arise in inflamed non-lymphoid tissues. This is reported for diverse sites in chronic inflammatory autoimmune diseases, such as the central nervous system (CNS) in multiple sclerosis (8), the kidney in systemic lupus erythematosus (SLE) (9), the inflamed synovia in rheumatoid arthritis (RA) (10) or the salivary glands in Sjögren’s syndrome (11). By conferring survival advantages, such microenvironments can protect pathogenic PCs against immunosuppressive therapies and contribute to therapy resistance. Consequently, PC accumulation can support local antibody production, promoting inflammation (12–14), which in turn may modulate PC pathogenicity, e.g. through changes in the glycosylation profile of secreted Ig (15–17). In spite of these findings, our understanding of the biology of pathogenic PCs and their crosstalk with soluble and cellular hematopoietic and non-hematopoietic factors in inflammatory niches remains underexplored. Here, we will discuss the current knowledge of how different aspects of inflammation may change tissue microenvironments to promote PC differentiation, retention, survival and function. We will elucidate commonalities and differences between conventional and pathogenic niches and discuss how communicating niche components may reciprocally shape each other.

## Pathogenic PCs - marked by unique immune-phenotypic markers?

Despite their well-documented presence, PCs residing in inflamed tissues are poorly characterized. For instance, a gain or loss of PC marker expression is found in myeloma PCs (18–20). Such knowledge could be valuable for selective targeting approaches in chronic inflammatory diseases. Even in a healthy context, universal approaches to identify PCs are limited (21–24), with CD138 and CD38 being the most salient, however not entirely specific, immune-phenotypic markers. Although additional markers, such as TACI, Sca-1, Ly6C, SLAMF7 or CD98, have been defined in mice, their function and regulation in inflammatory conditions remains unclear (21, 23–27). Considering, for example, that type-I interferon or TNF can modulate Sca-1 expression (21, 28, 29), reveals an important knowledge gap. Differential CD43 and B220 expression in renal PCs, compared to BM and spleen, in lupus-prone mice further suggests that localization may affect PC phenotype (9). Furthermore, there is a need for markers to selectively identify pathogenic PCs. Recently, exclusive Gp49B expression was found in PCs of lupus-prone mice (30). While their role in disease pathology needs to be determined, high co-expression of CD39 and CD326 in murine BM-resident PCs was linked to possibly protective anti-dsDNA IgM production (26). High PC heterogeneity constitutes another obstacle hampering selective targeting. Although this could reflect various PC differentiation stages (31), there is a need to define pathogenic or regulatory PCs in an inflamed context.

## PC development and compartmentalization in chronic inflammation

Various factors, such as cellular source, developmental path and final destination, may shape PC heterogeneity. In autoimmune disease, extra-follicular, GC-dependent and T-independent pathways can feed into the pool of short- and long-lived (LL) PCs (32–38). Allelic risk factors (34), chronic antigen exposure and local nutrients and cytokines (39) may influence the choice. PC differentiation in chronically inflamed non-lymphoid organs, adds another level of complexity. Here, inflammation can drive the formation of a) tertiary lymphoid structures (40, 41) found in influenza-infected lungs (42) or organ rejection post-transplantation (43), b) of lymphoid infiltrates without GC-like structure, e.g. in synovium of RA patients or salivary glands of Sjögren’s syndrome (44, 45) or, c) lympho-myeloid infiltrates with or without follicular structure, found in lupus nephritis (38, 46, 47). It is still unclear whether these sites arise in response to inflammation or harbor specific (auto)immune responses to local tissues (38), whether emerging PCs feed the same pool as PCs from lymphoid tissues (48) and, whether preferential homing to the BM versus nearby niches in inflamed organs occurs. In murine lupus, studies indicate a dispersion of autoreactive PC, showing highest frequencies in the kidney compared to the spleen and BM (9, 13). In accordance with data from mucosal immunity, this argues against strict compartmentalization. Here, a fraction of murine IgA^+^LL-PCs does not recirculate after formation, but persists in the lamina propria, while some join the LL-PC pool in the BM (3). This may ensure stable, long-term protective humoral immunity. However, in autoimmunity and considering that inflammatory niches may newly arise, but also vanish with dissolving inflammation, this may favor the persistence of pathogenic PCs and refractory disease. Conversely, immunization of lupus-prone mice shows that PC-specificity in inflammatory niches is not restricted to self-antigens that may compete with conventional PCs immigrating from lymphoid organs (49, 50). Hence, factors driving dynamics, compartmentalization and competition of PCs for limited numbers of inflamed and non-inflamed niches, should be addressed in further studies.

## Concerted actions of inflammation, metabolic status and hypoxia in PC niches

Inflammation may alter microenvironmental nutrient supply/demand ratios and shares an interdependent relationship with hypoxia (51, 52), that may impact the biology of PCs and their niches. For instance, hypoxia increases plasmablast generation from human memory B cells (53) and could affect myeloma PC pathophysiology by altering metabolic pathways (54, 55). Also in non-malignant PC, it may be speculated, that PCs metabolically adapt to such microenvironmental changes on a molecular level. For example, LL-PCs were shown to engage autophagy and pyruvate-dependent respiration (56–60) and are typically located in the physiologically hypoxic BM milieu supporting PC longevity (39, 53, 61, 62). Data from multiple myeloma support such scenarios, revealing high HIF1-α/HIF2-α expression (63), driving critical interactions with BM cells (64). The link between hypoxia and inflammation may even have a wider significance, as HIF-1 pathways and NF-κB signaling are linked, with the latter supporting PC survival (51, 65–68). Hence, inhibition of these pathways, as well as targeting metabolic vulnerabilities of PCs, may be attractive therapy approaches in multiple myeloma and chronic inflammatory diseases.

## Infiltrating immune cells may shape inflammatory niches

In homeostasis, the CXCL12-CXCR4-axis controls the access of PCs to “exit points” such as the BM, and their spatial organization within designated domains located there (7, 69), which are enriched in survival factor-producing cells. These include various immune cells, such as megakaryocytes, eosinophils, dendritic cells (DCs), monocytes, myeloid progenitors or regulatory T cells that may redundantly foster the survival of co-localizing PCs through soluble and membrane-bound factors (70–75). For instance, DCs are in frequent contact with PCs and may promote survival *via* CD80/86, binding CD28 on the PC surface (76–78). Meanwhile, secretion of soluble factors, such as APRIL and IL-6 represents another means of promoting PC survival. They are particularly secreted by myeloid precursors, as well as eosinophils, the role of which in BM niches still needs clarification (70, 73, 79–82). Similarly, immune cells may shape inflammatory niches, PC longevity and functionality. Here prevailing signals may possibly induce quantitative and qualitative changes in accessory immune cell infiltration and differentiation, providing putative therapeutic targets. In murine lupus nephritis, PCs populate the tubulointerstitium (9), an area where DCs and macrophages reside in both a homeostatic and inflamed state (83–85). Kidney-infiltrating macrophages/monocytes in human nephritis have been reported to represent a major source of IL-6, TNF-α and APRIL (86, 87). Furthermore, TNF-α can activate NF-kB signaling (88), which in turn increases the expression of BAFF (89) and CD80 (90, 91). Interestingly, several lupus-prone strains display elevated CXCL12 in inflamed kidneys, and hyper-expression of CXCR4 on PCs (92). This may augment PC homing to inflamed kidneys and co-localization with accessory immune cells. Likewise, in both mice and humans, LL-PC were found in the inflamed CNS within survival niche-like tissue areas, characterized by an up-regulation of APRIL and CXCL12 and the adhesion molecule VCAM-1 (8, 93–95). Moreover, PCs were found in RA synovial tissues, where recruitment of APRIL-producing neutrophils and macrophages was reported (96). Also, both epithelial and infiltrating mononuclear cells in the salivary glands of Sjögren’s patients can be potent producers of CXCL12 and IL-6 (11). In chronic inflammation, further molecules may become relevant for PC homing, such as CXCR3, sensing pro-inflammatory CXCL9, 10 and 11. Due to the interferon-induced up-regulation of this axis, it may gain additional importance in RA, SLE and other inflammatory diseases (49, 97–100). However, incomplete effects in blocking this axis in mice with established lupus nephritis, suggest redundant roles of further, not yet identified pathways (101). Also, whether these guide PCs towards niches with a unique composition is not yet clear. Moreover, it needs to be better understood how recruited PC themselves adapt to their new environments and shape their organization. As discussed below, studies suggest bi-directional communication and mutual influences between PCs and other niche components.

## Stromal cell impact on humoral immunity in chronic inflammation

Stromal cell function goes beyond a merely architectural role for tissue integrity and homeostasis. Instead, stromal cells orchestrate tissue microenvironments and immunity and may importantly shape PCs and their niches (102, 103). In the murine BM, about 80% of PCs are in direct contact with stromal cells (104), however, these stromal populations can be highly heterogeneous (105–107). On a molecular level, PC survival is mediated by direct crosstalk between PCs expressing adhesion molecules VLA-4 and LFA-1, interacting with VCAM-1 and ICAM-1 in stroma (62, 108–111). Additionally, soluble factors released by stromal cells, including CXCL12, IL-6, BAFF, and APRIL, are important players in this interaction (62, 104, 112–114). Extracellular matrix components, such as laminin-ß1, could particularly participate in the maintenance of mouse BM IgG-secreting PCs (59). Immunomodulatory stromal cell characteristics and their ability to incite, chronify and uncouple tissue inflammation to distinct anatomical sites (115–121), suggests a role in shaping inflammatory PC niches. On the other hand, inflammation may cause epigenetic modifications and induce ‘inflammatory memory’ at the level of tissue stroma (122), which would fit the concept that persistently activated stromal cells provide a ‘fertile soil’ for the incitement and spread of chronic inflammatory diseases (122–131). Similar to homeostatic niches, stromal and epithelial cells in lupus nephritis (132, 133) and salivary glands in Sjögren’s syndrome (11) were identified as CXCL12 producers, some even produced IL-6 in response to anti-dsDNA antibodies (134). Moreover, chronic inflammation changed immunoregulatory properties of mesenchymal stromal cells (MSCs) which adopt common characteristics of a senescent phenotype able to exacerbate inflammation (135–141). In murine lupus nephritis, MSCs contributed to formation of tertiary lymphoid structures by acting as lymphoid tissue organizer cells (142). Under homeostatic conditions, MSCs could inhibit immunoglobulin production in mouse PCs through CCL2 secretion (143) and suppress excessive B cell maturation by inhibiting BAFF secretion (144). Also, human MSCs could impair B cell differentiation and subsequent Ig secretion and impair their chemotaxis by down-modulation of CXCR4 and CXCR5 (145). This indicates that stroma may exert a determinant role for PC function. Regardless of the niche and in concert with other cellular and soluble participants, they may fulfil the needs for PC longevity in a redundant fashion. Stroma remodeling in PC niches by inflammatory stimuli needs to be investigated further as it may open up new options for treatment of chronic inflammatory diseases.

## Communication in inflamed niches may be bi-directional

The viewpoint that PCs only secrete copious amounts of antibodies is increasingly challenged. Their ability to produce cytokines, miRNAs and express co-stimulatory molecules (69) suggests they may communicate in a bidirectional, possibly tripartite way, with other cells. Interestingly, in SLE, even autocrine APRIL production by PCs was reported, which may drive their survival in an autocrine loop (146). Modulating and paracrine effects are best documented for regulatory and IL-10-expressing PC (147–149). While in infectious and cancerous microenvironments IL10^+^PCs dampened anti-microbial (147, 150) and anti-tumor immunity (150–152), they exert protective effects in autoimmunity (148, 153, 154). Gut-homing PCs can secrete further cytokines such as TNF-α, TGF-β or IL-17 that may drive disease pathology also in inflamed niches (155–157). Moreover, PCs may indirectly increase cytokine levels, supporting their own survival through interaction with other immune cells: as specified above, they could induce IL-6 in DCs *via* CD28/CD80/CD86 (76). Aged PCs may even modulate myelopoiesis, reportedly in an IL-10- and TLR-dependent manner (158, 159). This is relevant, since myeloid cells and progenitors are important producers of PC survival factors (71, 73, 74, 87) and may provide a positive-feedback loop. Thus, mutual influences between inflammation, immune aging and TLR-signaling may not only be relevant in inflamed PC niches, but also influence the survival of pathogenic PCs in homeostatic niches, which warrants further study.

## Conclusion

In conclusion, data suggest that the reciprocal relationship between communicating components in inflamed PC niches may propagate inflammation and disease progression, creating a vicious cycle. More data are required to substantiate these assumptions, identify key players and understand the dynamics in these functional units. Moreover, it is important to define differences and commonalities between conventional and pathogenic PCs and their niches and to clarify whether molecular structures at different sites are shared, although the cells providing these structures are tissue-specific. The identification of defined signatures would offer new perspectives for the design of specific targeting approaches, especially in patients resistant to conventional therapies, thereby saving protective PCs.

## Author contributions

All authors listed have made a substantial, direct, and intellectual contribution to the work and approved it for publication.
